# A Statistical Damage Constitutive Model Based on the Weibull Distribution for Alkali-Resistant Glass Fiber Reinforced Concrete

**DOI:** 10.3390/ma12121908

**Published:** 2019-06-13

**Authors:** Zhende Zhu, Cong Zhang, Songsong Meng, Zhenyue Shi, Shanzhi Tao, Duan Zhu

**Affiliations:** 1 Key Laboratory of Ministry of Education for Geomechanics and Embankment Engineering, Hohai University, Nanjing 210098, China; 13851886169@163.com (Z.Z.); Mengsongsong_edu@163.com (S.M.); taoshanzhi@163.com (S.T.); 18852001281@163.com (D.Z.); 2 Jiangsu Research Center for Geotechnical Engineering Technology, Hohai University, Nanjing 210098, China; 3 College of Mining and Safety Engineering, Shandong University of Science and Technology, Qingdao 266590, China; shihongyue@126.com

**Keywords:** AR-Glass Fiber, tensile failure, damage evolution, Weibull distribution, constitutive equation

## Abstract

The addition of alkali-resistant glass fiber to concrete effectively suppresses the damage evolution such as microcrack initiation, expansion, and nucleation and inhibits the development and penetration of microcracks, which is very important for the long-term stability and safety of concrete structures. We conducted indoor flat tensile tests to determine the occurrence and development of cracks in alkali-resistant glass fiber reinforced concrete (AR-GFRC). The composite material theory and Krajcinovic vector damage theory were used to correct the quantitative expressions of the fiber discontinuity and the elastic modulus of the concrete. The Weibull distribution function was used and an equation describing the damage evolution of the AR-GFRC was derived. The constitutive equation was validated using numerical parameter calculations based on the elastic modulus, the fiber content, and a performance test of polypropylene fiber. The results showed that the tensile strength and peak strength of the specimen were highest at a concrete fiber content of 1%. The changes in the macroscopic stress–strain curve of the AR-GFRC were determined and characterized by the model. The results of this study provide theoretical support and reference data to ensure safety and reliability for practical concrete engineering.

## 1. Introduction

At present, concrete is one of the most widely used and indispensable engineering materials for large-scale infrastructure construction projects such as tunnels, roads, water conservancy projects, and marine engineering projects [1]. However, concrete is a multi-pore quasi-brittle material with high compressive strength, low tensile strength, high brittleness, low toughness, and poor impact resistance. Due to the increased use of concrete in construction, crack development has become the focus of increased attention [2,3]. Various research methods and techniques have been developed by many experts and scholars to improve the tensile properties, toughness, and ductility of concrete [4]. Alkali-resistant glass fiber reinforcement of concrete is a method for improving the performance of concrete that has been rapidly developed and widely applied in recent years.

Alkali-resistant glass fiber reinforced concrete (AR-GFRC) is a composite material composed of cement slurry, or concrete, as the base material and AR-glass fiber as the reinforcing material. Several studies have been conducted on cracks and aging in AR-GFRC. Soranakom and Bakhshi [5] investigated the dry shrinkage behavior of AR-GFRC using a combination of experiments and developed numerical calculation, theoretical models of the concrete strain history, and the prediction of crack width to determine the concrete tensile stress evolution and crack propagation. Bakhshi and Mobasher [6] studied the influence of glass fiber on the shrinkage of cracks in concrete and developed a water diffusion model for minimizing the development of cracks. Uniaxial tensile tests of AR-GFRC were conducted by Kasagani [7]; the orientation of the specimen’s fracture plane was observed with an optical microscope and the strength of the fiber material was determined. For the concrete cracks problem, a stochastic distribution method was proposed to determine the mechanical properties of micro AR-GFRC. Yildizel and Ozturk [8] demonstrated the important role of glass fiber in improving the tensile strength, energy absorption capacity, and crack propagation characteristics of composites. The durability of different AR-GFRC was investigated by Bentur and Bassat [9]; the aging effects on the flexural properties and the microstructure of the composites were determined in different environments and the test results were verified by scanning electron microscopy (SEM). Chandramouli, Rao, et al. [10] investigated the durability of concrete specimens with different fiber content using a rapid chloride ion penetration test; the effect of the glass fiber content on the concrete durability and strength was determined. As new techniques have been developed, nondestructive testing methods have been used to monitor the aging process of GFRC. The basic mechanism of nonlinear aging behavior of GFRC materials was described by Eiras and Bonilla [11]. A dynamic mechanical analysis (DMA), thermogravimetric analysis, SEM, and transmission electron microscopy were used to analyze polypropylene/polystyrene/glass fiber (PP/PS/GF) composites [12]. The tensile properties of glass fiber reinforced PP/PS blends were investigated in laboratory tests and the experimental results were compared with those of the Hui-shia model; a good agreement was found. X-ray diffraction (XRD) and SEM were used by Kwan et al. [13] to test the durability and permeability of AR-GFRC; the damage, strength, and ruptures were determined for different fiber dosages (0.6–2.4%). In addition, the crack formations, the minimization of cracks, and the aging of concrete were studied in references [14,15,16,17].

Many problems associated with crack development and aging in concrete have not been addressed in recent research studies and require further investigation. Crack occurrence and propagation are nonlinear types of behavior at the macroscopic scale, as has been demonstrated in previous research [4,7,9]. The mechanical properties of AR-GFRC have been described in previous studies but the damage evolution mechanism and failure mechanism of concrete at the microscopic scale have not been extensively investigated [10,11]. New methods and techniques have been used for the micro-analysis of concrete damage but the applicability and rationality of the model have not met the requirements of practical engineering [12,13]. Although prediction models, calculation methods, and stress–strain curves have been developed and/or described in recent research, concrete damage and different types of failure mechanisms and factors have not been discussed. The coupling of different factors has also been neglected and often resulted in a large deviation between the experimental and theoretical results.

In this study, the composite material theory and statistical damage theory were applied and the concrete discontinuity was investigated by revising the parameters associated with the concrete discontinuity and the elastic modulus. The Weibull distribution probability density function was used to derive an equation describing the statistical damage evolution of the AR-GFRC and a numerical feature method was used to determine the statistical parameters of the model. Subsequently, the proposed model was extended and validated based on the composite material’s elastic modulus, the fiber content, and a performance test of similar material to ascertain the applicability of the model and meet the engineering needs.

## 2. AR-GFRC Uniaxial Tensile Tests

### 2.1. Preparation of the AR-GFRC Specimens

#### 2.1.1. Basic Mechanical Parameters of the AR-Glass Fibers

In order to determine the effect of the AR-glass fibers on the mechanical properties of the concrete, Anti-Crak®HD/alkali-resistant glass fiber and Anti-Crak®HP/alkali-resistant glass fiber were selected as reinforcing materials in the uniaxial tensile test [18]. The mechanical parameters are shown in Table 1.

#### 2.1.2. Composition and Mix Ratio of the Concrete Matrix

The concrete matrix is composed of cement, sand, gravel, mineral powder, and admixture in certain proportions. In order to achieve the best performance and mechanical properties of the high-performance concrete, the mix ratio was obtained by using the ‘Full Calculation Method’, described by Mehta and Aitcin [19].

The concrete matrix contained the following materials in certain proportions: P.O 42.5 ordinary Portland cement, Wenhe middle sand from Tai’an (China), 5–20 mm continuous graded gravel, S95 grade slag mineral powder, and SM-IV polycarboxylic acid superplasticizer. The concrete specimens’ strength grade in the tensile test was C30. The specimen preparation was conducted in accordance with the laboratory specifications. The dimensions of the flat tensile test specimens were 600 mm × 600 mm × 60 mm and the specimen die was made of channel steel with dimensions of 60 mm × 40 mm × 60 mm. The mix ratios of the different fiber contents and lengths are shown in Table 2 and Table 3. Specimens with fiber volume contents of 0%, 0.5%, 1% and 1.5% and specimens of HD 6 mm and HP 12 mm with 1% fiber content were prepared.

### 2.2. Test Instruments and Test Scheme of the AR-GFRC

**Figure 1:**
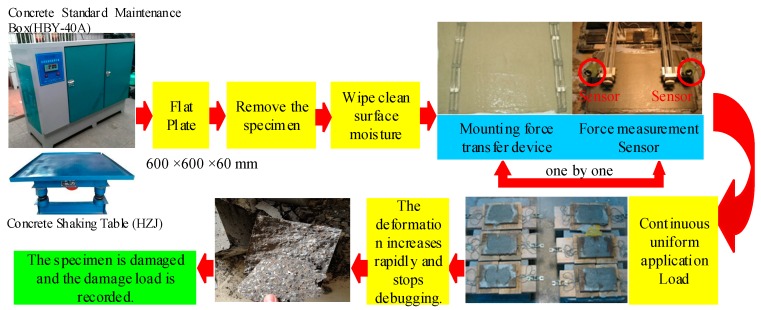
Test scheme of the alkali-resistant glass fiber reinforced concrete (AR-GFRC).

An HJW-60 concrete mixer (Kexing instrument equipment Co. Ltd., Cangzhou, China), HZJ concrete shaking table, HBY-40A concrete standard curing box, and YJ-22 electric measuring instrument (Zhaolong zhongke building instrument Co. Ltd., Cangzhou, China) were used in the tensile test. Three test specimens were selected for each group and the test results were accurate to 0.1 MPa during the test [20]. The test scheme is shown in Figure 1.

### 2.3. Test Results and Analysis of the AR-GFRC

#### 2.3.1. Macroscopic Crack Failure Patterns of the Specimens

**Figure 2:**
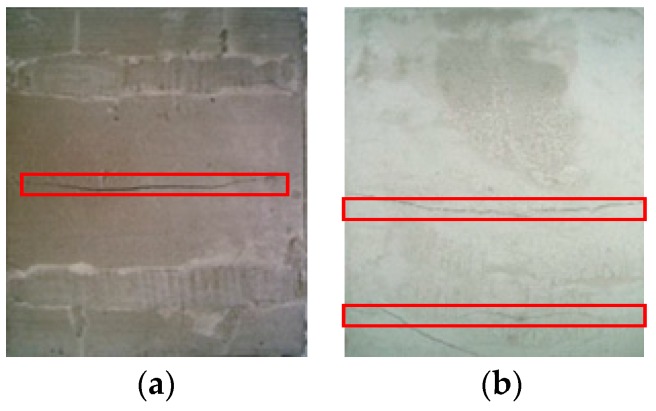
Crack morphology of ordinary concrete and AR-GFRC. (**a**) Ordinary concrete; (**b**) AR-GFRC.

For the plain concrete, once a crack appeared, the ultimate tensile strength was reached; the crack propagated until it penetrated the entire fault surface, as shown in Figure 2a. For the AR-GFRC, the fibers prevent crack opening at the interface of the cracks under a tension load. With increasing tension load, the fibers were pulled out, the cracks continued to expand, and the specimen was destroyed, as shown in Figure 2b.

As shown in Figure 2a,b, interface bonding force between cement and aggregate was weakened by the development of the cracks at the initial stage under a tensile load and a portion of the fracture energy was consumed. The bonding capacity of the concrete increased after addition of the AR-glass fiber and the fracture energy consumed by the cracks increased. Surface cracks gradually developed in the plain concrete with an increase in the external tension load. However, in the AR-GFRC, the cracks developed more slowly than in the plain concrete specimens and only a few small cracks appeared around the main cracks. This occurred because when a certain amount of fibers was added, the cohesion of the concrete was improved and the distribution of the AR-GFRC was random and unordered. The cracks were randomly distributed and expanded. During this process, the fiber content is the main factor influencing the cohesion of the material.

#### 2.3.2. The Influence of the Fiber Content

**Figure 3:**
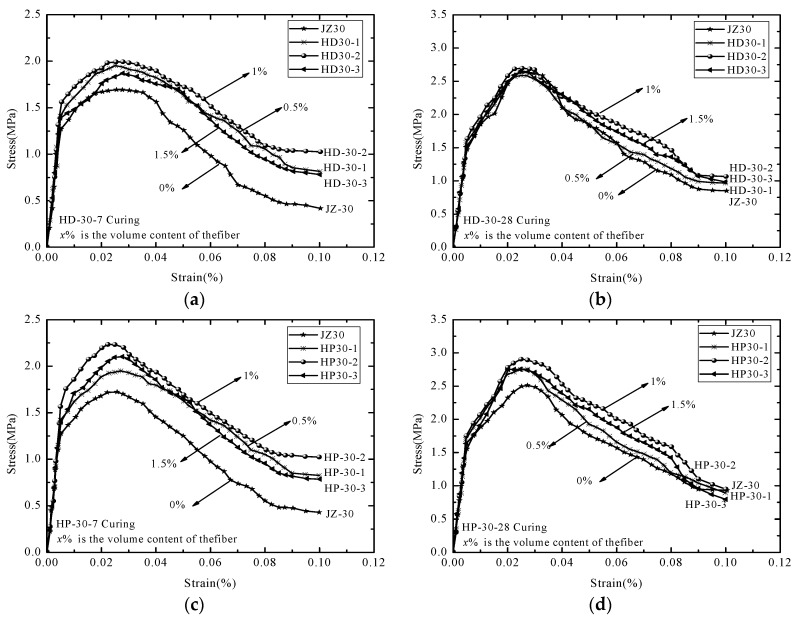
Tensile curves of AR-GFRC and ordinary concrete. (**a**) HD 7 d curing; (**b**) HD 28 d curing; (**c**) HP 7 d curing; (**d**) HP 28 d curing.

In order to investigate the effect of the fiber content on the tensile strength of the concrete, HD12 and HP12 AR-glass fiber were added to the concrete matrix. The strength grade was C30 and the volume content was 0%, 0.5%, 1%, and 1.5%. Using the control variable method, the tensile test of fiber concrete with different contents was carried out, and the tensile stress–strain curve of the AR-GFRC is shown in Figure 3. The tensile stress–strain curve represents a macroscopic reflection of the concrete’s tensile performance and parameters and of the characteristics of cracks’ appearance, development, damage accumulation, penetration, and failure of the concrete under a tensile load. As shown in Figure 3, the AR-GFRC specimens had higher peak strength and residual strength after cracking than the plain concrete (0% fiber content of JZ30). At the same strain, the AR-GFRC exhibited higher stress. The tensile strength was higher for the AR-GFRC than the plain concrete at AR-glass fiber contents of 0.5%, 1%, and 1.5%. However, the peak strength and tensile strength first increased and then decreased with increasing fiber content at a fiber content of 1%. The tensile strength and peak strength of the concrete were highest for standard curing for 7 d and 28 d. The peak strength of the HD and HP concrete with different fiber contents for 7 d and 28 d curing are shown in Table 4.

## 3. Development of the Statistical Damage Constitutive Model for AR-GFRC

In order to investigate the reinforcement and failure mechanism of the AR-GFRC, a constitutive equation of the damage evolution of the AR-GFRC is derived. At present, research on the damage of fiber reinforced concrete is mostly based on the composite material theory and the fiber spacing theory. However, the latter theory was put forward based on linear elastic fracture mechanics and is based on empirical results of strengthening tests of fiber reinforced concrete. The theoretical results are in good agreement with the experimental results for steel fiber reinforced concrete but there are large differences for AR-GFRC. Therefore, the damage constitutive model of the AR-GFRC is developed based on the composite material theory in this section.

### 3.1. Composite Materials Theory

**Figure 4:**
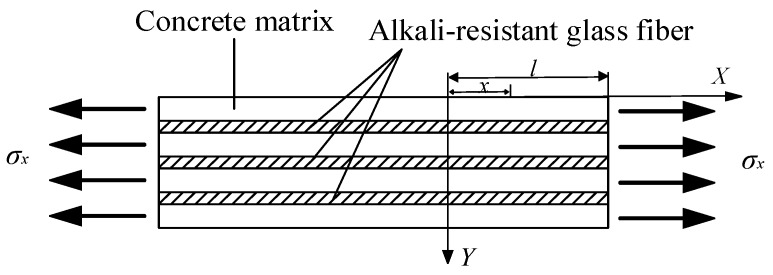
Tensile by effort of AR-GFRC.

In the composite material theory, the composite material is regarded as a two-phase matrix; the fiber is one phase and the matrix is the other phase. The mechanical properties of a composite material are affected by the matrix properties and fiber properties. In order to develop the constitutive model, the basic assumptions are as follows:

(1) The concrete matrix and AR-glass fiber are both isotropic linear elastic materials.
(2) The fibers stress direction and the distribution are parallel to the external tension load.
(3) When tension deformation occurs in the concrete, the deformation is the same for the fiber and the concrete and no relative sliding or dislocation occurs. The stress of the concrete is shown in Figure 4.

Based on the mechanical equilibrium equation and assumptions (1), (2), and (3), according to Saint Venant’s Principle, Equation (1) can be expressed as:

(1) $$\frac{\partial^{2}u}{\partial x^{2}}=-\frac{\sigma_{x}}{E_{fc}}$$

where *u* is the displacement in the direction of the *x* axis, σ*_x_* is the applied stress and *E_fc_* is the elastic modulus. Equation (1) can be expressed as:

(2) $$u=-\frac{\sigma_{x}}{2E}x^{2}+AX+B$$

**Figure 5:**
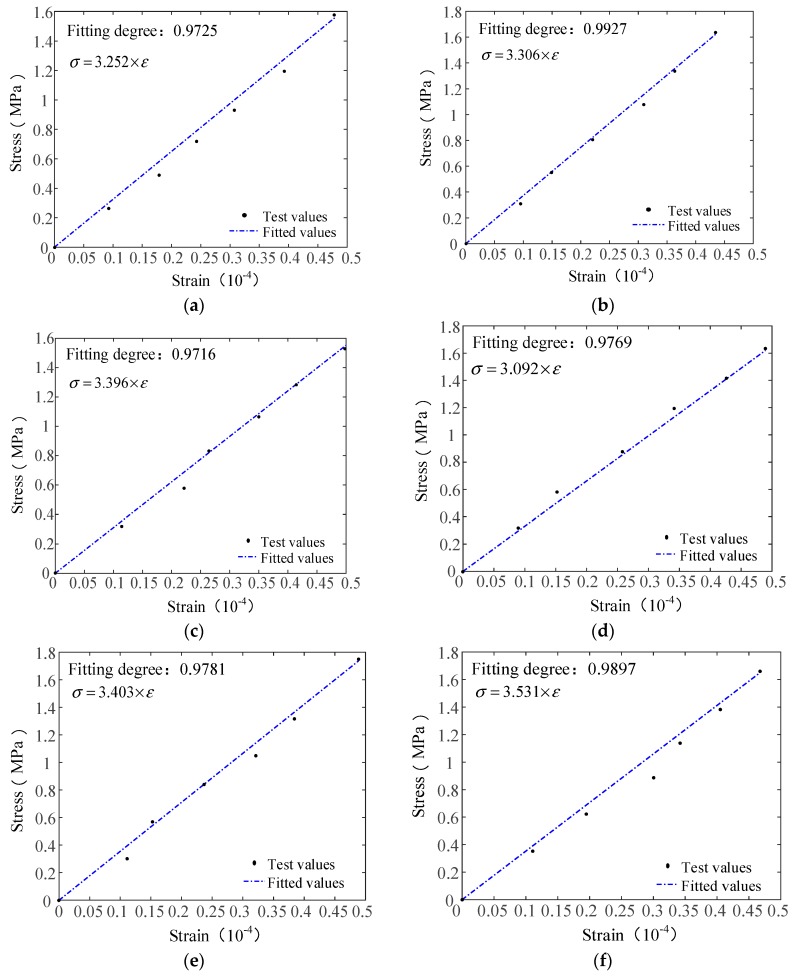
Comparison of the elastic modulus test curve and theoretical curve. (**a**) Elastic modulus fitting diagram of HD30-1; (**b**) Elastic modulus fitting diagram of HD30-2; (**c**) Elastic modulus fitting diagram of HD30-3; (**d**) Elastic modulus fitting diagram of HP30-1; (**e**) Elastic modulus fitting diagram of HP30-2; (**f**) Elastic modulus fitting diagram of HP30-3.

It can be obtained by boundary conditions *x* = 0, *x* = *l*:

(3) $$\sigma_{fc}=\frac{\sigma_{x}}{2}\left(l-2x\right)$$

where σ*_fc_* is the stress of the micro-body inside the material, as shown in Figure 5. The stress can be expressed as:

(4) $$\sigma_{fc}=\sigma_{f}V_{Ef}\frac{\left(l-2x\right)}{2}+\sigma_{m}V_{Em}\left(\frac{l-2x}{2}\right)$$

where σ*_fc_*, σ*_f_* and σ*_m_* are the stress of alkali-resistant glass fiber composites, glass fiber and matrix, and *V_E_* is the influence parameter of the volume modulus on the elastic modulus.

### 3.2. Fiber Discontinuity Correction

The AR-GFRC has a discontinuous and non-penetrating matrix. Therefore, assumption (2) must be modified. Previous research results have demonstrated that the continuous distribution of concrete fibers has a Weibull distribution. When calculating based on section method, the bonding length of the fiber is considered as 0~0.5*l_f_* and taking the average value is $\lambda \Gamma \left(1+1/k\right)l_{f}$ (E=λΓ(1+1/k), where *λ* is a proportional parameter, *k* is a shape parameter and Γ is gamma function). Where $\eta_{e}=\lambda \Gamma \left(1+1/k\right)$, the mechanical equilibrium equation of a single fiber is:

(5) $$\pi d_{f}{\bar{\eta }}_{l}\eta_{e}l_{f}\tau =\sigma_{f}\frac{1}{4}\pi d_{f}^{2}$$

where $\eta_{l}=4{\bar{\eta }}_{l}\eta_{e}$,

(6) $$\sigma_{f}=\eta_{l}\frac{l_{f}}{d_{f}}\tau$$

where *l_f_* and *d_f_* are the fiber length and fiber diameter, *τ* is the average value of the bonding stress between the fibers and the concrete matrix, *η_e_* is the fiber length mean coefficient obtained by Weibull function and ${\bar{\eta }}_{l}$ is the effective bonding length coefficient of the fibers. Equation (6) is substituted into Equation (2):

(7) $$\sigma_{fc}=\left(\sigma_{m}V_{Em}+\eta_{l}\frac{l_{f}}{d_{f}}\tau V_{Ef}\right)\left(\frac{l-2x}{2}\right)$$

### 3.3. Modified Elastic Modulus of Composites

It is assumed that the cohesion *τ* between the fibers and matrix is proportional to the tensile strength of the AR-GFRC:

(8) $$\tau =\eta_{f}\sigma_{f}$$

where *η_f_* is the correlation coefficient of the matrix cohesive properties; the value depends on the fiber type, diameter, length, surface shape, and other factors. Equation (8) is substituted into Equation (7):

(9) $$\sigma_{fc}=\left(\sigma_{m}V_{Em}+\eta_{l}\eta_{f}\frac{l_{f}}{d_{f}}\sigma_{f}V_{Ef}\right)\left(\frac{l-2x}{2}\right)$$

The composite elastic modulus *E_fc_* is the first derivative of σ*_fc_* to *ε_fc_*. The derivative of Equation (9) is obtained:

(10) $$\frac{d\sigma_{fc}}{d\varepsilon_{fc}}=\left(\frac{\partial \left(\sigma_{m}V_{Em}\right)}{\partial \sigma_{m}}\frac{d\sigma_{m}}{d\varepsilon_{fc}}+\eta_{l}\eta_{f}\frac{l_{f}}{d_{f}}\frac{\partial \left(\sigma_{f}V_{Ef}\right)}{\partial \sigma_{f}}\frac{d\sigma_{f}}{d\varepsilon_{fc}}\right)\left(\frac{l-2x}{2}\right)$$

where $\frac{dV_{Em}}{d\varepsilon_{fc}}=0,\frac{dV_{Ef}}{d\varepsilon_{fc}}=0,d\varepsilon_{fc}=d\varepsilon_{f}=d\varepsilon_{m}$. Equation (10) is deduced to the following:

(11) $$E_{fc}=\frac{l-2x}{2}E_{m}V_{Em}+\frac{l-2x}{2}\eta_{l}\eta_{f}\frac{l_{f}}{d_{f}}E_{f}V_{Ef}$$

In order to simplify Equation (9), we define $\eta_{\gamma }=\eta_{l}\eta_{f}$, where *η_γ_* is the correction coefficient of the AR-glass fiber elastic modulus in the composite and *α* is the length–diameter ratio of the fibers, that is *α* = *l_f_*/*d_f_*.

(12) $$E_{fc}=\frac{l-2x}{2}E_{m}V_{Em}+\frac{l-2x}{2}\alpha \eta_{\gamma }E_{f}V_{Ef}$$

### 3.4. Statistical Damage Constitutive Equation of the AR-GFRC

The AR-GFRC uniaxial tension can be divided into the undamaged and damaged evolution stage, in which the undamaged stage obeys Hooke’s law. For the damaged evolution phase, the uniaxial tension constitutive equation of the concrete is obtained based on the Krajcinovic Vector Damage Theory [21,22] and the Clausius–Duhem inequality:

(13) $$\sigma_{ij}\{\begin{array}{c} E_{fc}\varepsilon_{ij}\begin{array}{cc} & 0\leq \varepsilon \leq \varepsilon_{c} \end{array}\\ E_{fc}K\left[\begin{array}{ccc} 1+\left(C_{1}+C_{2}\right)D & \bar{\upsilon }+\frac{1}{2}C_{1}D & \bar{\upsilon }+\frac{1}{2}C_{1}D\\ \bar{\upsilon }+\frac{1}{2}C_{1}D & 1 & \bar{\upsilon }\\ \bar{\upsilon }+\frac{1}{2}C_{1}D & \bar{\upsilon } & 1 \end{array}\right]\varepsilon_{ij}\begin{array}{cc} & \varepsilon >\varepsilon_{c} \end{array} \end{array}$$

where $\bar{\upsilon }=\frac{\upsilon }{1-\upsilon }$, $K=\frac{1-\upsilon }{\left(1+\upsilon \right)\left(1-2\upsilon \right)}$, and *D* is the initial crack damage in the material. *C*_1_ and *C*_2_ are material constants. For AR-GFRC, *C*_1_ = 3.5–6, *C*_2_ = 0.35–0.7.

Based on assumption (3), the microcrack growth mainly occurs in the plane perpendicular to the tensile axis. According to the incremental formula, it can be concluded that:

(14) $$\left\{\begin{array}{c} d\sigma_{11}\\ 0\\ 0 \end{array}\right\}=E_{fc}K\left[\begin{array}{ccc} 1+\left(C_{1}+C_{2}\right)D & \bar{\upsilon }+\frac{1}{2}C_{1}D & \bar{\upsilon }+\frac{1}{2}C_{1}D\\ \bar{\upsilon }+\frac{1}{2}C_{1}D & 1 & \bar{\upsilon }\\ \bar{\upsilon }+\frac{1}{2}C_{1}D & \bar{\upsilon } & 1 \end{array}\right]\left\{\begin{array}{c} d\varepsilon_{11}\\ d\varepsilon_{22}\\ d\varepsilon_{33} \end{array}\right\}+E_{fc}K\left[\begin{array}{c} C_{2}\varepsilon_{11}+\frac{1}{2}C_{1}(\varepsilon_{22}+\varepsilon_{33})\\ \frac{1}{2}C_{1}\varepsilon_{11}\\ \frac{1}{2}C_{1}\varepsilon_{11} \end{array}\right]dD$$

That is:

(15) $$d\varepsilon_{22}=d\varepsilon_{33}=-\left(1-\nu \right)\left[\left(\frac{\nu }{1-\nu }+\frac{1}{2}C_{1}D\right)d\varepsilon_{11}+\frac{1}{2}C_{1}\varepsilon_{11}dD\right]$$

The hyperbola method is used to simplify the damage surface based on the Mohr–Coulomb criterion under uniaxial tension. At this time, the generated strain is the same as the damage evolution value. That is:

(16) $$f\left(\varepsilon_{11},D\right)=d\varepsilon_{11}-dD=0$$

Equation (16) is substituted into Equation (15) and the definite integral is obtained:

(17) $$\varepsilon_{22}=\varepsilon_{33}=\{-\left(1-\nu \right)\left[\left(\frac{\nu }{1-\nu }+\frac{1}{2}C_{1}D\right)\varepsilon_{11}+\frac{1}{2}C_{1}\varepsilon_{11}{}^{2}\right]$$

The initiation, development, and fusion of cracks in the AR-GFRC occur under tension. Fiber reinforced concrete is generally regarded as a series of defective micro-units in the statistical damage constitutive model [23]. The micro-unit strength criterion is expressed as follows:

(18) $$f\left(\sigma_{ij}\right)-k=0$$

where *f*(σ*_ij_*) is a stress function and *k* is related to the material parameters.

To ensure that *x* = (σ*_ij_*) and *p*(*x*) is the probability density distribution function of the micro unit, *N* is the total number of micro-units and *Np*(*x*)*dx* is the number of microelements destroyed in any interval [*x*, *x* + *dx*]. The damage variables of the AR-GFRC can be expressed as the ratio of the number of damaged micro units and the number of material micro-units. That is:

(19) $$D=\frac{\int_{0}^{x}Np\left(x\right)dx}{N}=\int_{0}^{x}p\left(x\right)dx$$

The development of damage or microcracks is not irregular and randomly distributed. Due to the influence of fracture energy and the difference in the elastic modulus of fiber and the concrete matrix, micro-crack development tends to be directional in the uncoordinated regions of deformation of materials. For the convenience of calculation, the contact surface position of the concrete matrix and fiber is equivalent to the distribution position of fiber. The deformation characteristics of the concrete, rock, and other materials and the microscopic heterogeneity of the mechanical properties of quasi-brittle materials follow the Weibull distribution [24,25]. Therefore, the strength of the concrete micro units is assumed to have a Weibull distribution. *p*(*x*) is expressed as:

(20) $$p\left(x\right)=\frac{m}{x_{0}}{\left(\frac{X}{x_{0}}\right)}^{m-1}\exp\left[-{\left(\frac{X}{x_{0}}\right)}^{m}\right]$$

where *m* and *x*_0_ are the statistical parameters of the Weibull distribution.

Equation (20) is substituted into Equation (19) to obtain the damage variable *D*:

(21) $$D=1-\exp\left[-{\left(\frac{X}{x_{0}}\right)}^{m}\right]$$

The strength of the micro-unit can be expressed based on the strength failure criterion of the Mohr–Coulomb micro unit:

(22) $$k=f\left(\sigma_{ij}\right)={\tilde{\sigma }}_{1}-{\tilde{\sigma }}_{3}-\left({\tilde{\sigma }}_{1}+{\tilde{\sigma }}_{3}\right)\sin\varphi =2c\cos\varphi =X$$

where *c* is the cohesive force of the concrete matrix and *φ* is the internal friction angle. According to the experimental mix ratio, *c* is 3.18 MPa and *φ* is 54.9°. Equations (15)–(17) and (21) are substituted into Equation (14):

(23) $$d\sigma_{11}=E_{fc}K\left\{\begin{array}{l} \left[1+\left(C_{1}+C_{2}\right)D+{\left(\frac{\upsilon }{1-\upsilon }\right)}^{2}+\frac{C_{1}D\upsilon }{1-\upsilon }+{\left(\frac{1}{2}C_{1}D\right)}^{2}\right]d\varepsilon_{11}\\ +\left[C_{2}-\upsilon -\frac{C_{1}D}{2}+\frac{C_{1}D\upsilon }{2}+\frac{C_{1}\upsilon }{2\left(1-\upsilon \right)}+\frac{1}{4}C_{1}{}^{2}D\right]\varepsilon_{11}d\varepsilon_{11}\\ -\frac{1-\upsilon }{2}C_{1}\varepsilon_{11}{}^{2}d\varepsilon_{11} \end{array}\right\}$$

That is:

(24) $$\begin{array}{l} k_{1}=1+\left(C_{1}+C_{2}\right)D+{\left(\frac{\upsilon }{1-\upsilon }\right)}^{2}+\frac{C_{1}D\upsilon }{1-\upsilon }+{\left(\frac{1}{2}C_{1}D\right)}^{2}\\ k_{2}=C_{2}-\upsilon -\frac{C_{1}D}{2}+\frac{C_{1}D\upsilon }{2}+\frac{C_{1}\upsilon }{2\left(1-\upsilon \right)}+\frac{1}{4}C_{1}{}^{2}D\\ k_{3}=\frac{1-\upsilon }{2}C_{1} \end{array}$$

Equation (24) can be simplified as follows:

(25) $$d\sigma_{11}=E_{fc}K\left(k_{1}d\varepsilon_{11}+k_{2}\varepsilon_{11}d\varepsilon_{11}-k_{3}\varepsilon_{11}{}^{2}d\varepsilon_{11}\right)$$

By defining the integral of Equation (25), we obtain:

(26) $$\sigma_{11}=E_{fc}K\left(k_{1}\varepsilon_{11}+\frac{k_{2}}{2}\varepsilon_{11}{}^{2}-\frac{k_{3}}{3}\varepsilon_{11}{}^{3}\right)$$

When the AR-GFRC is damaged and destroyed, the tensile capacity of the concrete matrix will be lost. However, the tensile crack resistance on the damaged fracture surface persists until the fibers are pulled out or are broken [26,27]. Therefore, the tensile strength of the fibers on the damaged surface is defined as:

(27) $$\sigma_{11}=E_{fc}K\left(k_{1}\varepsilon_{11}+\frac{k_{2}}{2}\varepsilon_{11}{}^{2}-\frac{k_{3}}{3}\varepsilon_{11}{}^{3}\right)+E_{f}S_{f}\varepsilon_{f}$$

where *S_f_* is the area ratio of the fibers on the damaged surface, *ε_f_* is the strain of the broken fibers, i.e., the elongation at the breakpoint. The statistical damage constitutive model of the AR-GFRC under tension can be obtained from Equation (13) and Equation (27).

(28) $$\sigma_{11}=\{\begin{array}{c} E_{fc}\varepsilon_{11}\begin{array}{cc} & 0\leq \varepsilon \leq \varepsilon_{c} \end{array}\\ E_{fc}K\left(k_{1}\varepsilon_{11}+\frac{k_{2}}{2}\varepsilon_{11}{}^{2}-\frac{k_{3}}{3}\varepsilon_{11}{}^{3}\right)+E_{f}S_{f}\varepsilon_{f}\begin{array}{cc} & \varepsilon >\varepsilon_{c} \end{array} \end{array}$$

The damage factor equation is:

(29) $$D=1-\exp\left[-{\left(\frac{X}{x_{0}}\right)}^{m}\right]$$

### 3.5. Determination of the Constitutive Model Parameters

Among the tensile constitutive model parameters of the AR-GFRC, the mechanical parameters of the concrete, such as *l_f_*, *d_f_*, *E_m_*, *E_f_*, *C*_1_, and *C*_2_, are determined by the characteristics of the AR-GFRC and can be obtained by tests. The others are statistical parameters, such as the two Weibull distribution parameters *m* and *x*_0_, which need to be determined based on the concrete tension and compression test curves. At present, the methods to determine the statistical parameters include linear fitting [28], the peak point method [29,30], the inversion analysis method [31], and non-linear regression [32,33]. In this section, the Weibull parameters are calculated and determined by the numerical feature method. The equation is:

(30) $$\frac{\Gamma^{2}\left(1+1/m\right)}{\Gamma \left(1+2/m\right)}=\frac{{\bar{x}}^{2}}{{\bar{x}}^{2}+S^{2}}$$

(31) $$x_{0}=\frac{\bar{x}}{\Gamma \left(1+1/m\right)}$$

where Γ is a function of Γ; $\bar{x}$ and *S* are the average strength and standard deviation of the AR-GFRC, respectively. That is:

(32) $$\bar{x}=\frac{1}{n}\sum_{i=1}^{m}\sigma_{i},\;\;S^{2}=\frac{1}{n-1}{\sum_{i=1}^{n}\left(\sigma_{i}-\bar{x}\right)}^{2}$$

The scale parameter *m* can be determined by Equation (31) and the shape parameter *x*_0_ can be obtained by using *x*_0_ in Equation (30).

## 4. Verification of the Statistical Damage Constitutive Model for AR-GFC

### 4.1. Determination and Verification of the Elastic Modulus of the Constitutive Model

The elastic modulus of the AR-GFRC consists of the elastic moduli of the undamaged part and damaged part; the test value of the elastic modulus of the AR-GFRC can be obtained from the concrete tensile test data. The elastic modulus of the proposed constitutive model can be calculated using Equation (12) and is then compared with the elastic modulus obtained from the test; this provides parameter information for the theoretical stress–strain curve. The experimental and constitutive theoretical stress–strain curve fitting is shown in Figure 5. The parameters required for calculating the elastic modulus are shown in Table 5.

The experimental and theoretical stress–strain curves before the damage evolution stage are well fitted. The experimental results of the elastic modulus of the AR-GFRC are close to the model values, which validates the uniaxial tensile constitutive model. The results demonstrate that the derivation process of the constitutive model is accurate and reasonable. The results in Figure 5a–f show that the theoretically derived elastic modulus is consistent with the elastic modulus values obtained from the experiment for the different fiber contents. In the elastic nondestructive stage, the theoretical and experimental stress–strain curves are well fitted and the fitting degree is larger than 0.93. Therefore, the theoretically derived elastic modulus is used as the benchmark parameter for model verification and it is suitable to conduct the fitting verification of the whole test curve and the theoretical curve.

### 4.2. Tests and Constitutive Model Verification of the Concrete with Different Fiber Contents

**Figure 6:**
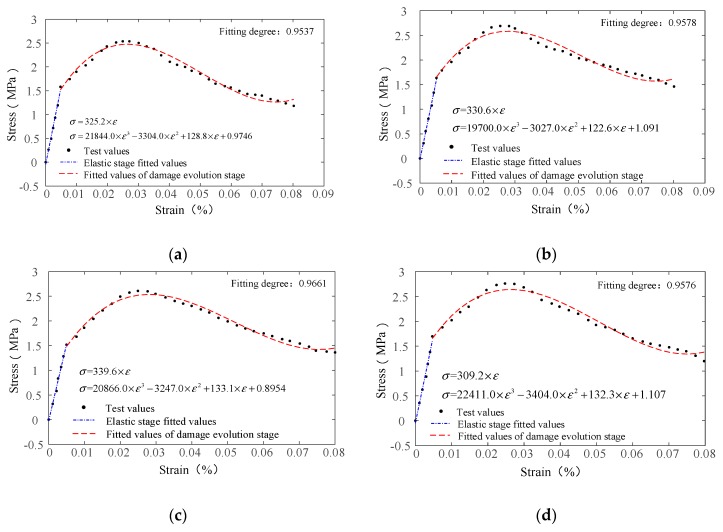

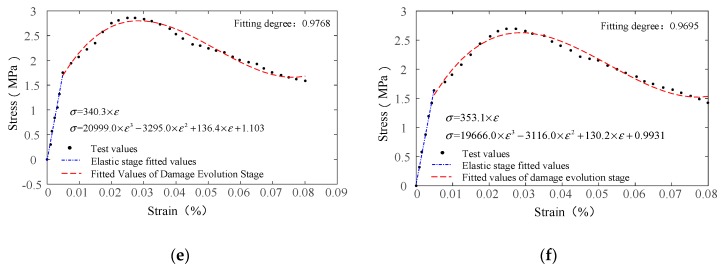
Comparison of test curves and theoretical curves. (**a**) Data fitting diagram of 0.5% HD; (**b**) Data fitting diagram of 1.0% HD; (**c**) Data fitting diagram of 1.5% HD; (**d**) Data fitting diagram of 0.5% HD; (**e**) Data fitting diagram of 1.0% HD; (**f**) Data fitting diagram of 1.5% HD.

The tensile test data of the HD and HP concrete with different fiber contents are used to verify the damage constitutive model of the AR-GFRC. In order to determine several test parameters in the constitutive model, six groups of HD and HP concrete test datasets with different fiber contents after 28 d of standard curing are used for the verification. The Poisson ratio of the concrete specimens was 0.3, the elastic modulus was the theoretical elastic modulus (as described in Section 4.1), and the other parameters used for validating the constitutive model are shown in Table 6. The comparison of test curves and theoretical curves is shown in Figure 6.

The results in Table 6 and Figure 6 indicate that as the fiber content increases, the model parameter *m*, which was obtained by the numerical feature method, shows an increasing trend, whereas the parameter *x*_0_ shows a decreasing trend. The stress–strain curves of the concrete specimens with different fiber contents obtained from the experiment are in good agreement with the stress–strain curves of the theoretical constitutive model prior to the peak section, although there are slight differences at the peak. These are mainly attributed to the individual differences of the specimens, that is, the maximum resistance of the test curves depends on the different fiber contents on the fracture surface. The experimental tensile strength is larger than the theoretical tensile strength but the theoretical constitutive curves and the experimental data curves are well fitted after the peak.

### 4.3. The Extension and Verification of the Constitutive Model for Similar Fiber Materials

**Figure 7:**
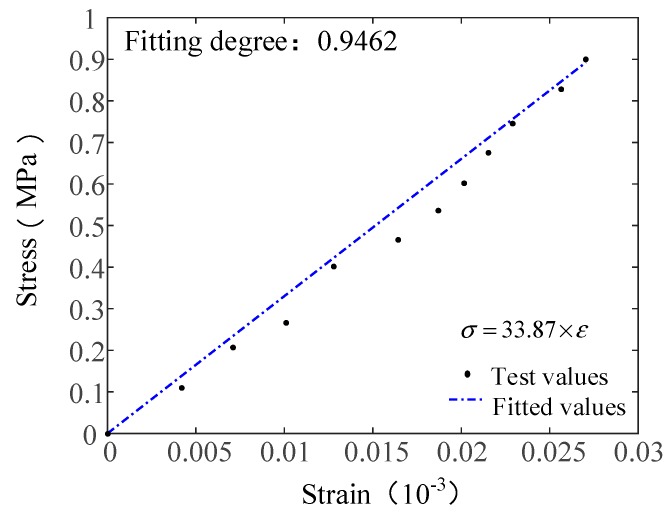
Comparison of the elastic modulus test curve and theoretical curve.

The extension and validation of the proposed constitutive model were determined by using experimental data for PP fibers as described in reference [34]. PP fibers and the C30 concrete matrix were used in the test; the specific parameters are shown in Table 7 and the experimental results are shown in A9. The mix ratio of the fiber concrete matrix is shown in Table 8. A plate test was used to measure the concrete’s tensile properties in reference [34]. The specimen had a prism shape with dimensions of 100 mm × 100 mm × 300 mm; the loading test was carried out after 28 d of curing and the loading rate was 0.02 mm/min. The data acquisition and loading control were conducted using computers. The method used to verify the elastic modulus was the same as described in Section 4.1. The theoretical elastic modulus value was 33.87 GPa and the fitting degree was 0.9462. Figure 7 shows the fitted curve for the theoretical and experimental results of the elastic modulus in Reference [34].

**Figure 8:**
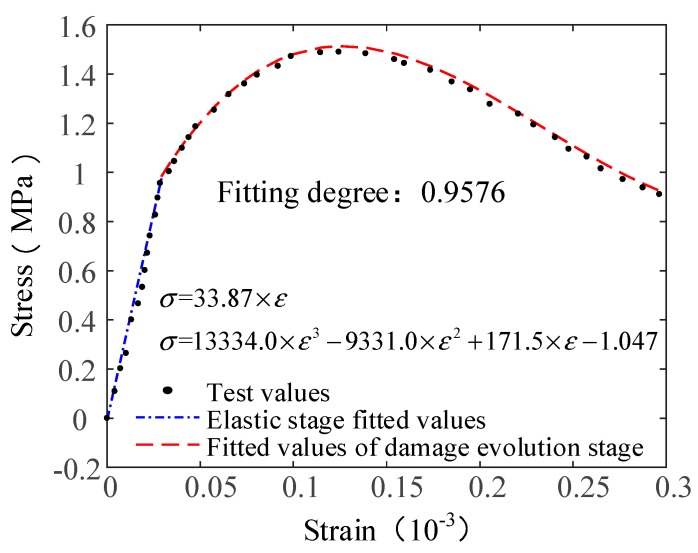
Comparison of the test curves and theoretical curves.

The results show that the elastic modulus of the PP fiber reinforced concrete obtained from the proposed method (Equation (12)) is close to the elastic modulus value obtained by the test. The trend before the damage evolution stage is consistent and a good fit was obtained. The fiber contents were 6% in group A9 and the verification method was the same as that described in Section 4.2. The Poisson ratio of the specimen was 0.32. The model parameters of the constitutive model are shown in Table 9. The comparison of the experimental and theoretical results is shown in Figure 8.

The results indicate that the stress–strain evolution of the concrete with the different fiber contents can be accurately described by the proposed damage constitutive model of the AR-GFRC. The theoretical constitutive curve and the experimental data curve fit well before the peak and the peak strength and peak point values are similar. In addition, the range of fiber content used for the model verification was large and there are few differences in the fracture surface, which results in a small difference in the maximum tensile strength between the experimental and theoretical results. Furthermore, the theoretical and experimental stress–strain curves are in good agreement after the peak. This demonstrates that the proposed statistical damage constitutive model of the fiber reinforced concrete is well suited for characterizing the stress–strain curve of the AR-GFRC using the tensile test data of the PP fibers.

In summary, the statistical damage constitutive model of the AR-GFRC is based on the composite material theory and Krajcinovic vector damage theory. It is assumed that the concrete consists of a series of isotropic elastic microarray elements and that the probability distribution of the micro-element failure follows a Weibull distribution. However, in practical applications, the interface between the concrete matrix and fiber is not isotropic and the derived constitutive equation may not describe the actual conditions accurately. Yet, lateral damage in concrete does not occur in an indoor uniaxial tension test. Therefore, the actual stress–strain of the concrete can be determined by the proposed constitutive model in the simplest form. Different fiber contents and the elastic modulus of the composite materials can be used to determine the meso-damage evolution and failure mechanism of the AR-GFRC and of the concrete in practical engineering applications. The results provide important practical and reference data to ensure safety and reliability for concrete engineering.

## 5. Conclusions

(1) Indoor flat tensile tests of AR-GFRC were conducted and the macro-crack failure modes of the plain concrete and AR-GFRC specimens were analyzed to determine the occurrence and development of cracks in the material. The peak strength of different types of concrete under standard curing was quantitatively analyzed to determine the influence of the fiber content on the tensile strength of the concrete. It was determined that the tensile strength and the peak strength were highest at a fiber content of 1%.
(2) The composite material theory and Krajcinovic vector damage theory were applied to modify the equations related to the fiber discontinuity and elastic modulus. The Weibull distribution function was used to derive the equation of the elastic modulus of the fiber reinforced concrete composite and the statistical damage constitutive model of the AR-GFRC was developed. The statistical parameters of the model were determined using the numerical feature method.
(3) The constitutive equation was validated based on the elastic modulus and the different fiber contents obtained from indoor uniaxial tensile tests. The validation results indicated a good fit of the theoretical and experimental results of the elastic undamaged stage and damage stage with a fitting degree larger than 0.93. Tensile test data of PP fibers made of similar materials were used to extend and validate the proposed constitutive model using the same validation method. The results showed a fitting degree larger than 0.92 for the theoretical and experimental stress–strain curve before the peak value. The statistical damage constitutive model of the AR-GFRC provides reference data and theoretical support to ensure the safety and stability of concrete structures.

## Figures and Tables

**Table 1:** Basic mechanical parameters of different varieties of fibers.

Type	Length (mm)	Equivalent Diameter (um)	Fracture Strength	Elongation at Break (%)	Modulus (GPa)	Melting Point (°C)
HD	6/12	30	1700	3.6	60	1580
HP	6/12	30	1700	3.6	60	1580

**Table 2:** Mix ratio of C30 concrete slab with different fiber content.

Number	Cement	Sand	Stone	HD	HP	Water	Admixture
JZ30	370	758	1047	0	0	185	2.0%
HD30-1	370	758	1047	0.5	0	185	2.0%
HD30-2	370	758	1047	1.0	0	185	2.0%
HD30-3	370	758	1047	1.5	0	185	2.0%
HP30-1	370	758	1047	0	0.5	185	2.0%
HP30-2	370	758	1047	0	1.0	185	2.0%
HP30-3	370	758	1047	0	1.5	185	2.0%

**Table 3:** Mix ratio of C30 concrete slab with different fiber length.

Number	Cement	Sand	Stone	Fiber Contents	Water	Admixture
JZ30	370	758	1047	1.0	185	2.0%
Cem-FIL60-12	370	758	1047	1.0	185	2.0%
Cem-FIL60-18	370	758	1047	1.0	185	2.0%
HD-6	370	758	1047	1.0	185	2.0%
HP-12	370	758	1047	1.0	185	2.0%

**Table 4:** Mix ratio of C30 concrete slab with different fiber length.

Type	Time							
	7 d				28 d			
	0%	0.5%	1%	1.5%	0%	0.5%	1%	1.5%
HD	1.68	1.92	2.09	1.89	2.64	2.69	2.73	2.61
HP	1.66	1.88	2.24	2.15	2.51	2.73	2.98	2.79

**Table 5:** Parameters required for calculation of the elastic modulus.

Number	*E_m_* (GPa)	*ρ_m_*	*E_f_* (GPa)	*ρ_f_*	*α*	*η_l_*	*η_f_*
HD30-1	30	99.5%	60	0.5%	400	0.1	0.15
HD30-2	30	99%	60	1.0%	400	0.1	0.15
HD30-3	30	98.5%	60	1.5%	400	0.1	0.15
HP30-1	30	99.5%	60	0.5%	400	0.1	0.15
HP30-2	30	99%	60	1.0%	400	0.1	0.15
HP30-3	30	98.5%	60	1.5%	400	0.1	0.15

**Table 6:** Material parameters of the constitutive model.

Number	*C*_1_	*C*_2_	*x*^0^(10^3^)	*m*
HD30-1	-4.4	0.44	9.2841	2.4
HD30-2	-3.77	0.59	6.958	3.9
HD30-3	-3.81	0.58	5.9	5.2
HP30-1	-4.51	0.42	10.5358	2.1
HP30-2	-4.02	0.50	7.1738	3.5
HP30-3	-3.58	0.49	5.8271	4.8

**Table 7:** Similar fiber material parameters.

Fiber Marking	Diameter (mm)	Length (mm)	Elastic Modulus (GPa)	Elongation at Break (%)
FF1	0.026	12	4.5	40
FF4	0.1	19	4.5	40
CF2	0.8	50	7.4	10

**Table 8:** Basic mix ratio of fiber concrete.

Test Marking	Fiber Type	Cement	Sand	Stone	Water	Fiber Contents	Sand Rate
A9	FF1+FF4+CF2	406	548	1221	207	6	23

**Table 9:** Material parameters of the constitutive model.

Number	*C*_1_	*C*_2_	*x*^0^(10^3^)	*m*
A9	3.0382	0.298	5.6742	4.9
